# Human T-Lymphotropic Virus-1 Associated With Adult T-Cell Lymphoma: A Case Report

**DOI:** 10.7759/cureus.73769

**Published:** 2024-11-15

**Authors:** Pauline Le Gatt, Margaux Pinana, Pierre Reimbold, Inès Boussen, Geraldine Lescaille, Juliette Rochefort

**Affiliations:** 1 Oral Surgery, Pitié-Salpêtrière Hospital, Paris, FRA; 2 Pathology and Laboratory Medicine, Pitié-Salpêtrière Hospital, Paris, FRA; 3 Hematology, Pitié-Salpêtrière Hospital, Paris, FRA; 4 Odontology, Pitié-Salpêtrière Hospital, Paris, FRA; 5 Odontology, Health Faculty, Université Paris Cité, Paris, FRA

**Keywords:** adult t cell lymphoma, human t-cell lymphotropic virus type 1, oral manifestation, oral mucosal lesions, viruses

## Abstract

Human T-cell lymphotropic virus 1 (HTLV-1) was subsequently identified as the cause of adult T-cell leukemia/lymphoma (ATLL). While oral manifestations of this disease have been documented, they remain poorly described in the literature.

We present the case of a 32-year-old patient who exhibited facial and oral swelling in the upper jaw. Pathological examination confirmed a diagnosis of T-cell lymphoma, with immunohistochemical staining revealing HTLV-1 positivity. Following further diagnostic evaluation, the patient was classified as having stage IV HTLV-1-positive adult T-cell lymphoma. The patient underwent chemotherapy and bone marrow stem cell transplantation, which led to remission of both the oral lesion and the systemic disease.

This condition can be highly aggressive, depending on the subtype and stage at diagnosis. To date, only five similar cases have been reported in the literature, with a generally poor survival rate. Therefore, early recognition is crucial for timely management and improved outcomes.

## Introduction

General information

Lymphomas, first described by Thomas Hodgkin in 1832 [1], are defined as heterogeneous cancers resulting from the clonal proliferation of lymphocytic cells at different stages of maturation [2]. They are commonly divided into two categories: Hodgkin lymphomas and non-Hodgkin lymphomas [3]. Hodgkin lymphomas are characterized by the presence of giant, bilobed nucleus cells derived from B lymphocytes, known as Reed-Sternberg cells [4]. The most recent lymphoma classification corresponds to the 5th edition of the World Health Organization (WHO) classification of hematologic malignancies, published in 2022, which describes six categories, including myeloid tumors and proliferations, histiocytic/dendritic tumors, B-cell lymphomas and lymphoproliferations (grouped into five families), T-cell and NK-cell lymphomas and lymphoproliferations (grouped into three families), chorion-derived lymphoid tissue tumors, and genetic syndromic tumors [5,6]. Oral lymphomas represent 3% of all lymphomas and are the third most common cancer found in the oral cavity. Some of them may be induced by viruses known as oncogenic viruses, with the main ones being Epstein-Barr virus, human herpesvirus 8, and human T-cell lymphotropic virus 1 [2].

Clinical manifestations and diagnostic methods

Common general symptoms include fatigue, fever above 38 °C, night sweats, weight loss of more than 10% of total body weight, generalized pruritus, lymph node pain upon alcohol consumption, and unexplained inflammatory syndromes [7]. Biopsy is the key diagnostic procedure for oral lymphoma, performed at the site of the oral lesion with two samples: the first fixed in formalin and embedded in paraffin, and the second in a dry tube with Michel's solution for immunohistochemical analysis [8].

Therapy

The therapeutic strategy depends on the type of lymphoma, its stage, and the patient's risk factors. It is decided after a Multidisciplinary Tumor Board meeting [7]. Several treatment options are available [2,7], including chemotherapy, immunochemotherapy or targeted therapies, radiotherapy, and hematopoietic stem cell transplantation [9]. Chemotherapy can be administered orally or by vein, in which case, an implantable chamber is required. This is the most frequently used treatment, particularly for stages III or IV [9].

Immunochemotherapy or targeted therapies involve the use of monoclonal antibodies, which can be combined with conventional chemotherapies. Radiotherapy involves the use of ionizing radiation to destroy cancer cells in a localized manner, often in conjunction with chemotherapy for stages I and II, particularly in the gums. Hematopoietic stem cell transplantation, either autologous using the patient's own stem cells or allogeneic using stem cells from a compatible donor. Finally, patients with indolent, localized lymphomas presenting no symptoms may be advised to refrain from treatment [9]. 

Given the limited research on their localization in the oral cavity, we aim to present a clinical case highlighting this rarely diagnosed entity in the oral cavity.

## Case presentation

Case history and examination

**Figure 1:**
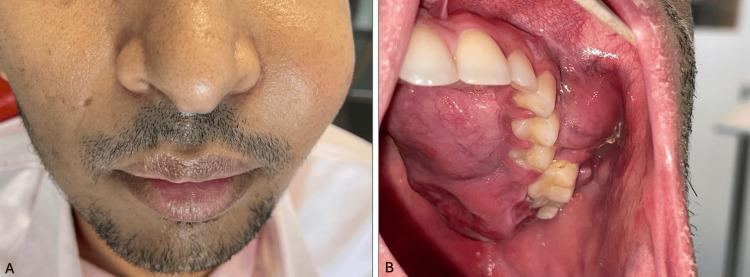
Left upper cheek swelling in a 32-year-old patient (A) Exobuccal view; (B) Endobuccal view showing a bluish 6 cm swelling on the left maxillary vestibular and palatal region adjacent to teeth 23 to 27, with an ulceration near tooth 26

We report the case of a 32-year-old patient with no prior medical history, no ongoing treatments, and no known allergies. His family history included a mother with diabetes and a sister with adult T-cell leukemia. The patient presented to the emergency department with a left maxillary mass that had been progressing for one month (Figure 1A).

Clinical examination revealed facial asymmetry with a left exobuccal swelling, along with a bluish palatal nodule measuring 6 cm adjacent to teeth 23 to 27, accompanied by ulceration (Figure 1B). This was associated with dental mobility, pain in the teeth and sinuses, and left-sided epistaxis.

The patient also reported a 7 kg weight loss over the past 3 months but did not experience fatigue, fever, or night sweats.

Differential diagnosis and investigations

Additional Examinations and Clinical Investigations

**Figure 2:**
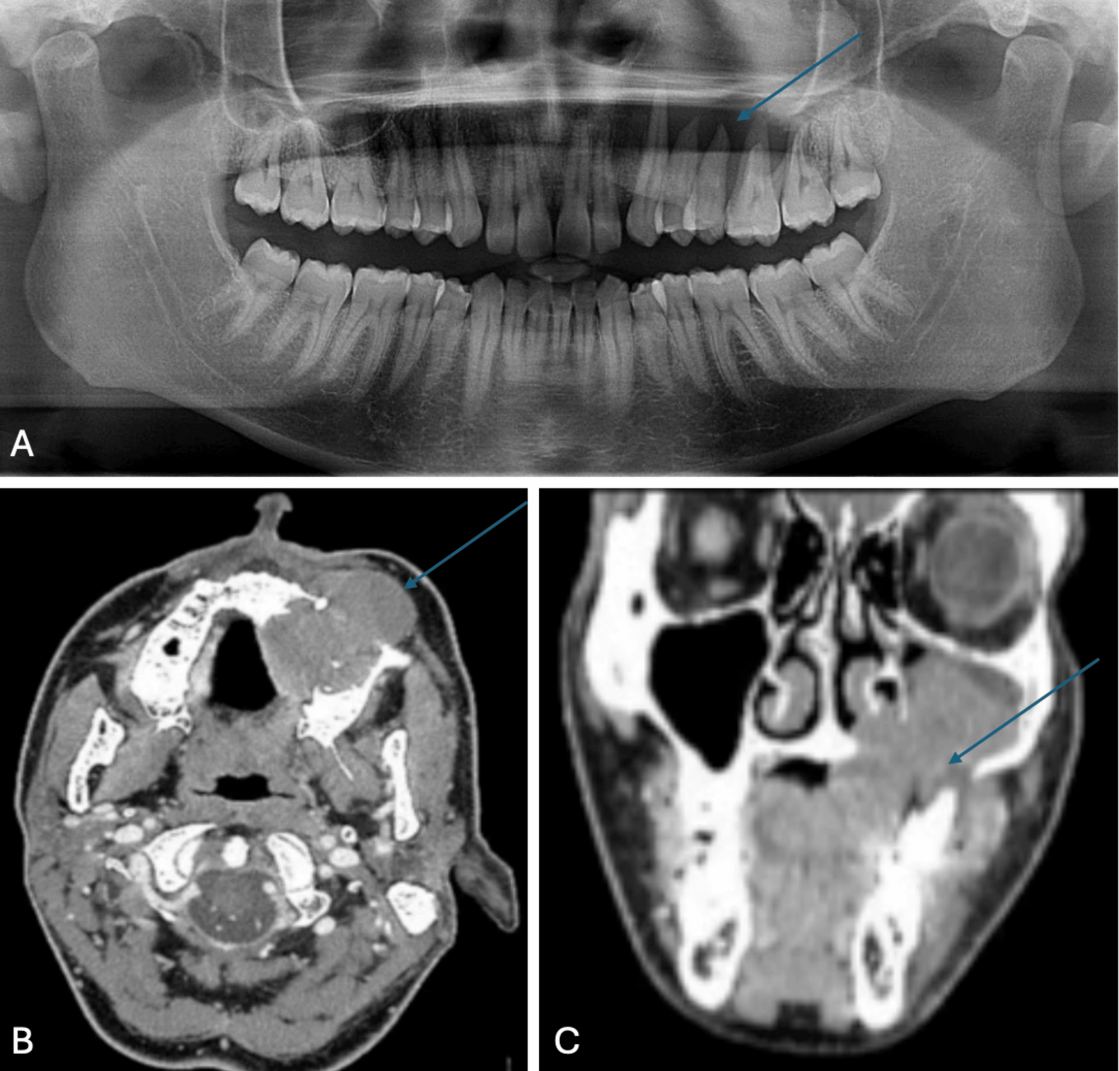
Additional imaging examinations (A) Panoramic radiograph of the patient revealing bone lysis in the second quadrant adjacent to the swelling at the level of teeth 23 to 27; (B) Axial slices from the cervicofacial CT scan showing a large tissue mass invading the left maxillary sinus and bone; (C) Coronal slices from the cervicofacial CT scan showing a large tissue mass invading the left maxillary sinus and bone

Radiographic examination with an orthopantomogram revealed bone lysis in the second quadrant (Figure 2A). Magnetic resonance imaging (MRI) showed a tissue mass centered on the maxillary sinus with bone lysis of its nasal floor and lateral walls, as well as ipsilateral lymphadenopathy in groups Ib and II, and contralateral group II lymph nodes.

The initial cervicofacial CT scan revealed a mass involving the entire left sinus and maxilla, measuring 47 x 35 x 50 mm in diameter with lysis of the nasal floor and lateral walls of the left maxillary sinus (Figures 2B, 2C).

Diagnostic Hypotheses

Based on this clinical presentation, our diagnostic hypotheses included oral lymphoma or a malignant lesion such as squamous cell carcinoma or sarcoma.

Biopsy and Pathological Examination

**Figure 3:**
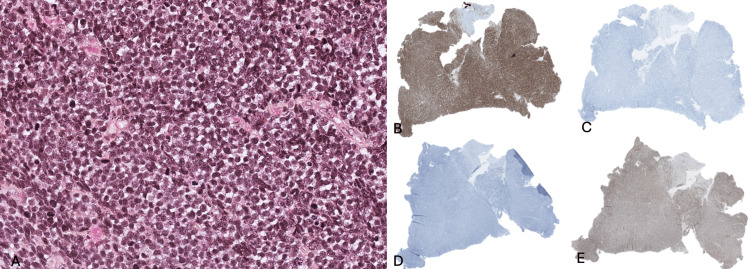
Additional examinations through anatomopathological analyses (A) Histological section stained with hematoxylin and eosin showing numerous lymphocytic cells (dark pink) associated with apoptotic bodies (light pink); (B) Immunohistochemical sections showing positive staining (in brown) for CD5 markers; (C) Immunohistochemical sections showing negative staining for CD7 markers; (D) Immunohistochemical sections showing negative staining for CD20 markers; (E) Immunohistochemical sections showing positive staining (in brown) for CD25 markers

A biopsy was performed to guide the diagnosis, with two samples taken: one fixed in formalin and the other in a dry tube with Michel's solution for immunohistochemical analysis. Microscopic examination revealed a diffuse lymphomatous proliferation, occupying the entire sample, consisting of monotonous cells with medium to large, rounded nuclei. Mitoses and apoptotic bodies were also observed (Figure 3A). Immunohistochemical analysis showed positive staining for CD5 (Figure 3B) and negative staining for CD20 (Figure 3C), along with the loss of CD7 expression (Figure 3D) and positive staining for CD25 (Figure 3E). These immunohistochemical markers are specific for adult HTLV1+ T-cell lymphomas [10].

Staging Assessment

Given this histological result, a staging assessment was conducted. The complete blood count was unremarkable, but blood phenotyping showed a circulating T-lymphocyte population. HTLV1 serology was positive, as was hepatitis B virus serology. The positron emission tomography (PET) scan revealed an intensely hypermetabolic lesion centered on the left maxilla, as well as a hypermetabolic right hilar lymph node. The lumbar puncture did not show any additional lymphomatous proliferation.

Outcome and follow-up

Diagnosis

Based on all these test results, the final diagnosis of stage IV adult HTLV1+ T-cell lymphoma (maxillary, nodal, and blood involvement) was made.

Treatments Administered

An initial treatment of four cycles of methotrexate-CHOEP (cyclophosphamide, doxorubicin, etoposide, vincristine, and prednisone) and two cycles of CHOEP were implemented. The patient subsequently received monthly cycles of Holoxan/VP16 (two milder chemotherapies) while awaiting an allogeneic stem cell transplant. The patient received a stem cell transplant from a family member and is currently in complete remission, with the latest follow-up in June 2024.

Progression and Follow-Up

**Figure 4:**
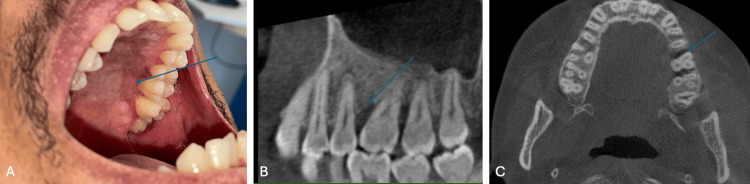
Clinical appearance after two years (A) Clinical view showing the absence of mucosal lesions; (B) Radiological sagittal view highlighting periodontal involvement at the level of teeth 26; (C) Radiological axial view highlighting periodontal involvement at the level of teeth 26

After two years of management, the patient no longer presents with oral lesions (Figure 4A). There are persistent pains not associated with mucosal lesions. Periodontal treatment was initiated to preserve teeth 26 and 27, which show residual bone loss (Figures 4B, 4C). Post-treatment PET scans after the second, fourth, and sixth cycles showed a complete metabolic response. A local pain management plan has been implemented. It is noteworthy that no teeth have been compromised in the management of this patient, despite the initial presentation of a large swelling.

## Discussion

Oral manifestations of this lymphoma remain rare in the literature, with cutaneous manifestations being more common [11]. Among the 116 patients identified in the literature with virus-induced oral lymphomas [12], only 5 cases involved adult HTLV-1+ T-cell lymphoma with oral manifestations. These findings align with the global prevalence of this virus, which ranges from 1% to 5%, depending on the region. Moreover, the countries where these five cases were reported - Brazil (South America), Japan, and Iran - are known to have a high prevalence of HTLV-1 infection [12-16].

The primary differential diagnosis for this lesion would be non-specific adult T-cell lymphoma, making HTLV-1 serology essential for diagnosis [17]. The prognosis of these lymphomas varies by subtype but can be very aggressive, emphasizing the importance of prompt management; thus, early diagnosis is critical [18].

Our clinical case involves a 32-year-old patient, which aligns with the literature, showing a male predominance (1.5 men to 1 woman) and an average age of 32, with all cases occurring between the ages of 30 and 45 [13-16]. Comparatively, adult T-cell lymphoma tends to present in a significantly younger population than other types of viral-induced oral lymphomas [12].

The clinical presentation of the ATL in our case was maxillary swelling, as in four out of five of the cases described in the literature, which also showed maxillary swelling, all associated with underlying bone lysis, and three of the five cases described in the literature have received chemotherapy as treatment and particularly CHOP (cyclophosphamide, doxorubicin, vincristine, and prednisone) as in our case [13-16].

A review of oral viral-induced lymphomas found that lesions typically progressed for an average of three months before the first biopsy [12]. In most cases, practitioners initially misdiagnosed these lesions as dental abscesses, leading to antibiotic therapy, tooth extraction, or debridement. Similar to our case, many patients delay seeking consultation despite noticing the growth of the lesion. These factors highlight the importance of educating practitioners and patients about the frequent diagnostic delays seen in oral lymphomas, whether virus-induced or not [12,19].

According to the literature, the prognosis for HTLV-1+ adult T-cell lymphoma shows a 2-year survival rate of just 20%, significantly lower than for other types of viral-induced oral lymphomas although this difference lacks statistical significance [12]. Most of these lymphomas were diagnosed at stage 4, and there was a significant difference in prognosis between stages 1-2 and 3-4 at diagnosis, with poorer survival rates for stages 3-4. This underscores the critical importance of early detection [12,19]​​​​​​.

This case raises the question of whether regular check-ups with an oral specialist should be recommended for known carriers of these viruses, particularly men over 20, as they appear to be more frequently affected. Additionally, systematic preventive follow-up could be considered for patients with family members or partners affected by these viruses, enabling earlier diagnosis.

## Conclusions

HTLV1-induced adult T cell lymphomas are rare, especially in the oral cavity, and clinical manifestation and localization are not specific. Our patient developed a mass in the upper maxillary gum around the teeth, which may look like a dental abscess. Indeed, many patients are initially misdiagnosed as having a dental infection, delaying the definitive diagnosis of lymphoma as in our case with an advanced-stage diagnosis.

Raising awareness of the latter could be beneficial not only for oral cavity specialists but also for treating physicians and even specialists in charge of monitoring HTLV1 patients, in order to reduce the diagnostic errancy of these pathologies in these patients.
